# Cerebral monitoring of very preterm infants with anterior cerebral artery resistive index and early NIRS

**DOI:** 10.55730/1300-0144.5577

**Published:** 2022-10-10

**Authors:** Mustafa Şenol AKIN, Fatma Nur SARI, Burak CERAN, Davut BOZKAYA, Esin OKMAN, Mihriban ALKAN, Evrim ALYAMAÇ DİZDAR

**Affiliations:** 1 Division of Neonatology, Department of Pediatrics, Ankara City Hospital, Ankara, Turkey; 2 Department of Radiology, Ankara City Hospital, Ankara, Turkey

**Keywords:** Prematurity, resistive index, NIRS

## Abstract

**Background/aim:**

The prediction of adverse conditions in the preterm neonatal brain might be improved by cerebral monitoring using combined measures of cerebral function, including oxygenation and blood flow parameters. To perform the consecutive measurements of the resistive index (RI) from the anterior cerebral artery (ACA) within the first week of life and to evaluate the association of these measurements with cerebral oxygen saturation (Csat) detected by near-infrared spectroscopy (NIRS).

**Materials and methods:**

This prospective cohort study enrolled very preterm infants, <32 weeks of gestational age, admitted to a tertiary neonatal intensive care unit. Csat levels were continuously monitored using NIRS for 72 h after birth. ACA RI measurements were obtained on the first, third, and seventh days of life by using transcranial Doppler ultrasound. These measurements were also compared between infants with and without unfavorable outcomes, including severe intraventricular hemorrhage (IVH) and early mortality.

**Results:**

A total of 96 preterm infants with Csat and ACA RI measurements were analyzed. Age at birth was 28.3 ± 1.9 weeks and birth weight was 1090 ± 305 g. The mean Csat of the infants was 77.1% ± 8.2% during the first 72 h of life. Mean ACA RI values were 0.76 ± 0.10, 0.75 ± 0.08, and 0.77 ± 0.08 on the first, third, and seventh days of life, respectively. RI on the first day of life was significantly higher in infants delivered by cesarian section than in those delivered vaginally (0.77 vs. 0.69; p = 0.017). Infants who died earlier had significantly higher ACA RI values on the first day than infants who survived beyond the first 7 postnatal days (0.83 vs. 0.76; p < 0.001).

**Conclusions:**

There was no association between ACA RI and Csat in the early period of life. ACA RI values on the first postnatal day might be significant for predicting early mortality in very preterm infants.

## 1. Introduction

Impaired brain function in newborns continues to be a dilemma. Newborns have a restricted repertoire of neurological signs and symptoms, and even with severe underlying pathological insults, they often exhibit only subtle ﬁndings. In particular, preterm infants face detrimental outcomes from conditions that affect brain function. Injury to the preterm brain can have a serious lifelong impact on the child, eventual adult, and family [1–3]. Therefore, efforts are required to prevent or minimize the effects of impaired brain function in this vulnerable population.

Cerebral monitoring by combining measures of cerebral function, including oxygenation and blood flow parameters, may improve the prediction of adverse conditions. The resistive index (RI) of cerebral arteries measured by transcranial Doppler ultrasonography is a noninvasive, radiation-free, safe, bedside neuroimaging modality that evaluates one aspect of cerebral blood flow [4].

Currently, measurement of the anterior cerebral artery (ACA) RI has become a common practice in some neonatal intensive care units (NICU) because it has been seen as a marker for perfusion of the brain, integrity of vascular compliance, and functionality of cerebral autoregulation [5]. There are data about the normal values of anterior cerebral artery RI in neonates. However, these measurements were obtained in a heterogeneous group of infants, and only a small number of very preterm infants were included; generally, a single observation was made at different time points [6–8]. There is a lack of evidence regarding the association between ACA RI and adverse neonatal outcomes. Near-infrared spectroscopy (NIRS), frequently used in preterm infants for monitoring cerebral oxygenation, reflects local perfusion and might provide real-time assessment of blood flow delivery [9]. However, little information exists about the dynamic changes in ACA RI during the first week of life and its association with cerebral oxygen saturation (Csat) during this critical postnatal period.

Our objective was to perform consecutive measurements of RI from the ACA within the first week of life and to evaluate the association of these measurements with Csat detected by NIRS in preterm infants younger than 32 weeks of gestation. Additionally, to assess if ACA RI or Csat is associated with severe IVH and early mortality.

## 2. Materials and methods

### 2.1. Study design and patient population

We conducted this prospective cohort study in a tertiary-level NICU between August 2019 and December 2019. Preterm infants, aged <32 weeks, were screened for eligibility. Infants with regional cerebral oxygen saturation monitored using NIRS during the first 72 h and serial cranial ultrasonography (cUS) imaging with RI measurements within the first week of life were enrolled. Infants with major congenital anomalies and hemodynamically significant patent ductus arteriosus (hsPDA) were excluded from the study. A few infants could not be included due to a lack of parental consent. Obstetric and neonatal data, including gestational age (GA), birth weight (BW), head circumference (HC), sex, mode of delivery, Apgar scores, antenatal steroid treatment, small for GA, preeclampsia, chorioamnionitis, and multiple pregnancies, were recorded prospectively. Data on severe IVH (grade > 2 according to the Papile classification) [10] and early mortality (within the first seven postnatal days) were also collected. The local ethics committee approved the study and written informed consent was obtained from the parents of each infant.

### 2.2. cUS protocol, Doppler measurement, and NIRS recording

**Figure 1:**
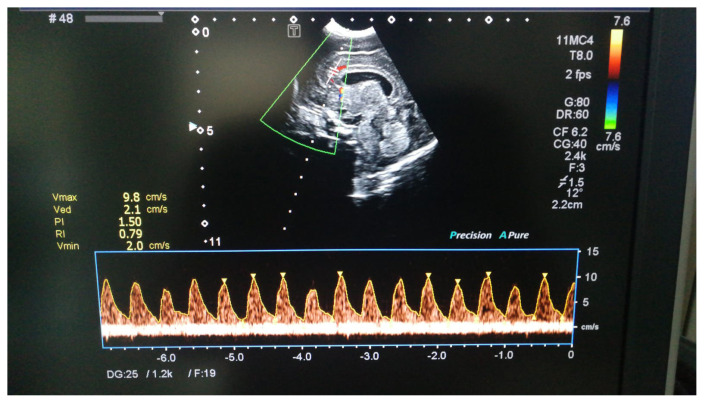
Resistive index (RI) is calculated from Doppler waveforms obtained from anterior cerebral artery with caliper placement in sagittal plane.

All Doppler studies were performed as part of the cUS examinations recommended by the national guidelines for very preterm infants on the first, third, and seventh day of life. A neonatologist with experience in neonatal neuroimaging recorded the Doppler measurements with a device (Toshiba Aplio^T^ 300, Canon Medical Systems, İstanbul, Turkey), which uses micro convex (CV) array transducers with frequencies between 8 and 10 MHz. The RIs were obtained from the anterior cerebral artery in the sagittal plane through the anterior fontanel. RI recordings were performed at the beginning of the examination to avoid any potential impact of continuous fontanelle stimulation on the intracranial hemodynamics. The neonatologist who obtained the Doppler measurements was blinded to the clinical condition of the infant. A consultant radiologist who was experienced in neonatal cUS performed routine cranial imaging and examined the sonogram of each Doppler measurement for image quality and appropriate caliper placement on the spectral waveform (Figure 1). RIs were automatically calculated from the maximum velocity waveforms using the following formula: (peak systolic velocity-end-diastolic velocity)/peak systolic velocity.

Cerebral oxygenation monitoring with NIRS is part of standard care within the first 72 h of life in our NICU for infants less than 32 weeks of gestation. Csat was monitored with an INVOS 5100C near-infrared spectrometer (Somanetics Corporation, Troy, MI). Neonatal NIRS sensors (OxyAlert NIRSensor, Covidien, Mansfield, MA, USA) were placed on the forehead of the infants. Csat was calculated from the differential signals obtained from these sensors and expressed as the venous-weighted percentage of oxygenated hemoglobin [oxygenated hemoglobin/total hemoglobin (oxygenated hemoglobin + deoxygenated hemoglobin)] [11]. NIRS recordings were performed continuously during the first 72 h of life. The recorded values were stored and used for data analysis after being transferred to a computer by a specialized program for the INVOS device.

### 2.3. Study outcomes

The primary outcome included the assessment of consecutive ACA RI measurements within the first week of life and the evaluation of the association between this ACA blood flow parameter and Csat. Secondary outcomes were the association of ACA RI and Csat with severe IVH and early mortality (within the first seven postnatal days).

### 2.4. Statistical analysis

Statistical analysis was performed using the SPSS software for Windows (version 20.0; IBM, Chicago, IL, USA). The distribution of continuous variables was confirmed using the Kolmogorov-

Smirnov or Shapiro-Wilk tests. Normally distributed data are presented as the mean and standard deviation, and other data are presented as the median and interquartile range (IQR). Categorical variables were presented as counts and percentages. The Mann-Whitney U test was performed to compare nonnormally distributed continuous variables. A paired t-test was performed to test the significance of pair-wise differences. The chi-square test or Fischer’s extract test was used to analyze categorical variables. Pearson’s and Spearmen’s correlation tests were used where appropriate. Receiver operating characteristic (ROC) curves were generated, and cut-off values to maximize the sensitivity and specificity of the ACA RI to predict mortality were chosen. In addition, intraobserver and interobserver agreement (interclass correlation coefficient) for RI measurements was evaluated in 10 randomly selected infants. Statistical significance was considered if the p-value was < .05. To show a correlation between ACA blood flow parameter and Csat, effect size |r| = 0.3, power (1-β error) = 80%, with an alpha error of 0.05, minimally sample size was calculated as 82.

## 3. Results

**Figure 2:**
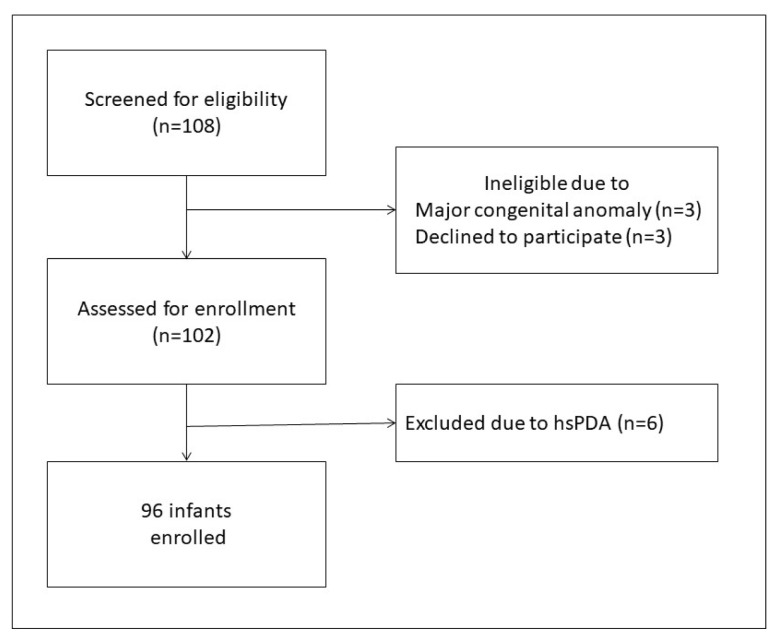
Flow chart.

Among the infants admitted to the NICU during the study period, 108 were eligible for inclusion in the study. We excluded 12 infants because of major congenital anomalies, lack of parental consent, or hsPDA. Data from 96 preterm infants were analyzed (Figure 2). Mean BW and GA for the total cohort were 1090 ± 305 g (range 375–1730 g) and 28.3 ± 1.9 weeks (range 24–31 weeks), respectively. Approximately one-third (n = 36) of the infants weighed <1000 g. Demographic and perinatal characteristics are summarized in Table.

**Figure 3:**
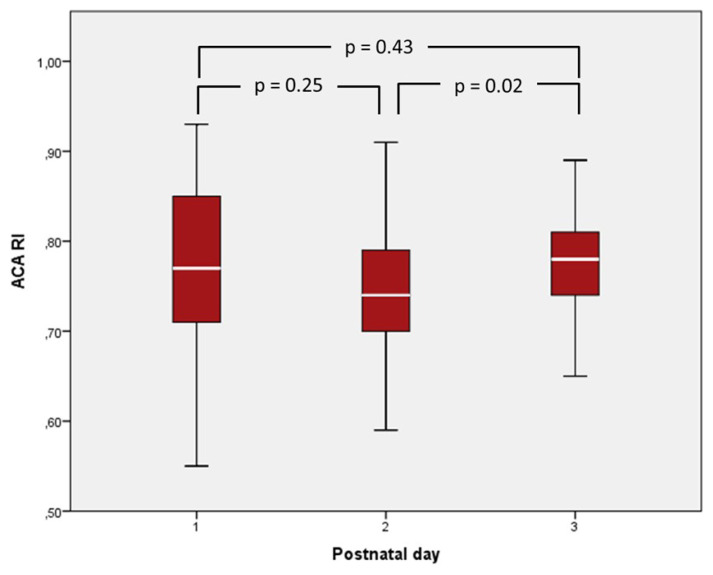
Anterior cerebral artery resistive index (ACA RI) values of the study infants.

Mean ACA RI values were 0.76 ± 0.10, 0.75 ± 0.08, and 0.77 ± 0.08 on the first, third, and seventh days of life, respectively. The seventh-day RI values were slightly higher than the values measured on the third day (p = 0.026) (Figure 3). Csat was continuously monitored for a median (IQR) duration of 64 (53–69) h. The mean Csat of the infants was 77.1% ± 8.2% during the first 72 h of life. There was no correlation between Csat and ACA RI values on the first, third and seventh days of life (1^st^ day: r = –0.03, p = 0.84, 3^rd^ day: r = –0.042, p = 0.80, 7^th^ day: r = 0.16 p = 0.41). There was also no correlation between RI values and gestational age or birth weight.

**Figure 4:**
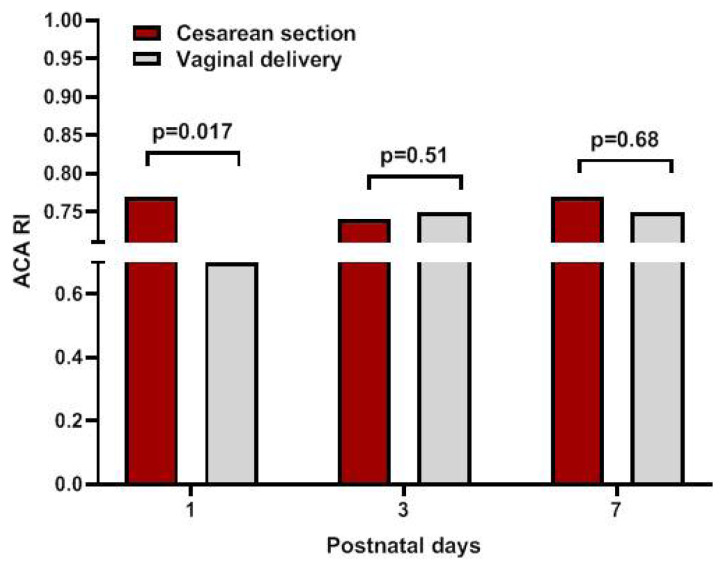
Anterior cerebral artery resistive index (ACA RI) values according to the delivery mode.

A comparison of RI values between infants delivered by cesarean section (C/S) and those delivered vaginally is shown in Figure 4. RI on the first day of life was significantly higher in infants delivered by C/S than in those delivered vaginally (0.77 vs. 0.69, p = 0.017); however, no difference was observed in subsequent measurements. There was no effect of sex on RI in our cohort. Girls and boys tended to have similar RI in consecutive measurements within the first week of life. Delayed cord clamping (DCC) was applied to 14 infants in the study group. There was no significant difference in consecutively measured ACA RI between infants with or without DCC.

ACA RI values also did not differ in infants born from multiple or single pregnancies and with maternal chorioamnionitis or not. Nine of the infants were SGA, and when compared to AGA ones no significant difference was observed at any Doppler measurements.

**Figure 5:**
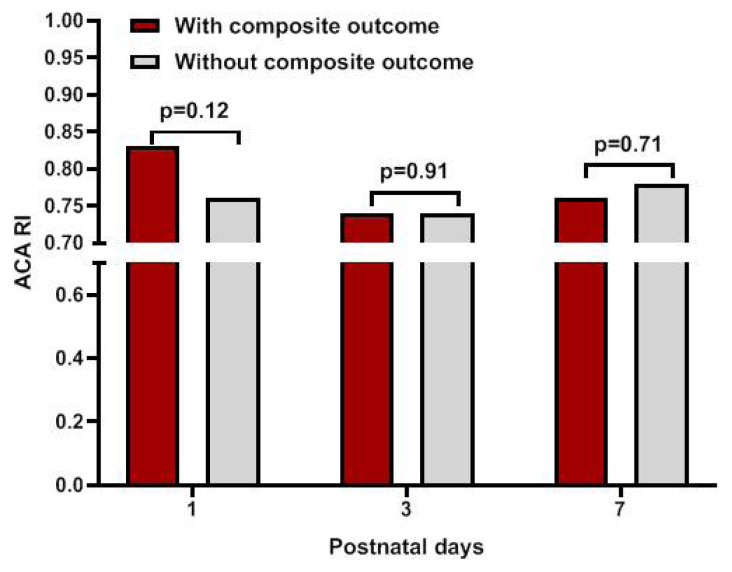
Anterior cerebral artery resistive index (ACA RI) values of infants with or without composite outcome.

Fourteen infants (14.5%) had the composite outcome of severe IVH (n = 6) or early mortality in the first seven postnatal days (n = 10). Two infants who died earlier also had severe IVH. There was no statistically significant difference in ACA RI values between the infants with and without composite outcome (Figure 5). Median (IQR) Csat did not significantly differ in infants with and without composite outcome [68% (64–84) vs. 77% (73–83); p = 0.28].

**Figure 6:**
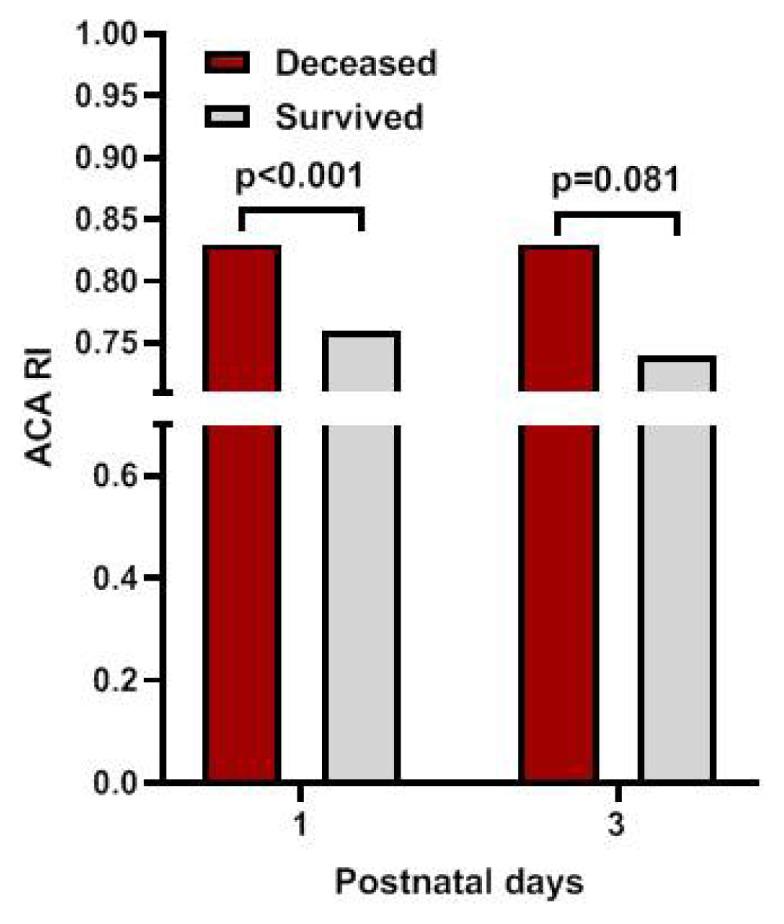
Comparison of anterior cerebral artery resistive index (ACA RI) values of infants who deceased and survived.

**Figure 7:**
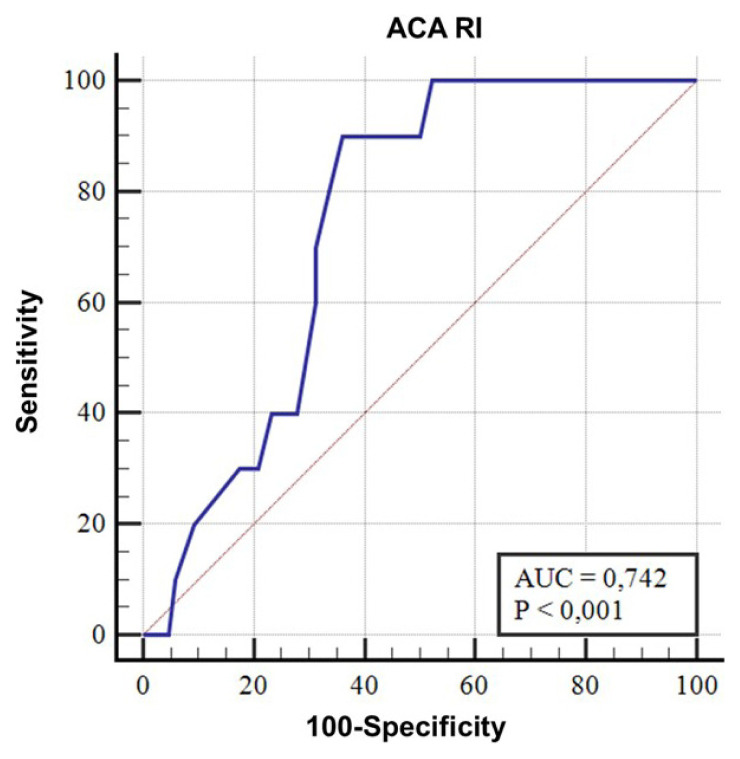
A ROC curve of the cut-off value for ACA RI on day 1 which predicts early mortality.

Of the total infants, six developed severe IVH (grade 3 IVH, n = 3; grade 4 IVH, n = 3) with a median age of diagnosis as day 3 (range 1–7). Two of these infants died on their 3^rd^ and 4^th^ days of life. Other causes of early death included asphyxia (n = 1), severe pulmonary interstitial emphysema (n = 1), early-onset sepsis (n = 3), and respiratory distress syndrome (n = 3). The median (IQR) age at early death was 3 (2–5) days. The infants who died earlier had significantly higher ACA RI values on the first day than those who survived beyond the first 7 postnatal days (Figure 6). A ROC curve was created which demonstrated a cut-off value for ACA RI on day 1 of >0.79 for predicting early mortality with an AUC of 0.74 (Figure 7). If ACA RI > 0.79, the sensitivity was 85% and specificity was 65% for early mortality. Furthermore, for both intra- and inter-observer reliabilities for RI measurements, a correlation coefficient of 0.98 was noted.

## 4. Discussion

In this study, we showed that ACA RI values measured in the first week of life were close to each other, however the index on the seventh day was slightly higher than that on the third day. No association was identified between ACA RI values and continuously monitored Csat in the early period of life. Infants with composite outcomes tended to have similar Csat and ACA RI values, while infants who died earlier had higher ACA RI on the first postnatal day. A higher ACA RI value on the first postnatal day in infants born by C/S reflects an association with delivery mode.

Neonatal neuroimaging plays an important role in both diagnosis and prognosis and may be useful in predicting neonatal outcomes [12–14]. Recently, the measurement of RI of ACA has become the subject of the studies, including those in newborns. Elmfors et al. [15] studied the RI of the pericallosal artery in more mature infants with a mean gestational age of 34.3 weeks ranging from 26 to 42 weeks. RIs are commonly measured on the first day of life. Compared to that study, we obtained higher RI values on the first day of life which might be due to the lower gestational age of our study infants. As gestational age decreases, RI increases [16, 17].

Consistent with Elmfors et al. [15], we found an association between delivery mode and ACA RI values. Infants delivered by C/S had higher RI values on the first day of life, and there were no differences in consecutive measurements. Thus, the absence of a difference in subsequent measurements proves the correlation between RI values and mode of delivery. However, it is difficult to comment on other perinatal conditions that could affect this finding.

Zamora et al. [18] determined ACA RIs at baseline and after brief compression to compare the variability in preterm and term infants younger than three months of age. T﻿he baseline ACA RI in very preterm infants was lower than the serially measured values seen in our study. However, postnatal age at first sonographic examination (mean ± SD: 7 ± 13.9 days for preterm infants) was later than that in our study. The delayed timing of the first ultrasound could explain the incompatibility of baseline measurements. The paper by Ecury Goosen et al. [19] reported the RIs of the cerebral arteries in very preterm infants throughout their NICU stay. Consistent with our study, they also obtained higher ACA RI values early after birth.

Decreased Csat has been associated with adverse neonatal outcomes [13, 20, 21]. We hypothesized that combining cerebral blood flow measurements with simultaneously monitored cerebral oxygenation might improve our understanding of the hemodynamic changes leading to unfavorable outcomes. Thus, we serially measured ACA RI and continuously monitored Csat at the critical period of postnatal life. Very little data exists regarding the association between Csat and ACA RI, which has been seen as an indirect measure of cerebral autoregulation. Recently, Altit et al. [14] found an association between Csat and ACA blood flow measurements in extremely low birth weight infants [14]. In contrast to our study, this was a retrospective review. Doppler values obtained from ACA were recorded on later days of the first week, and brief cerebral saturation, which was recorded starting 1 h before head US, continued until 1 h after cranial US. Differently, we showed no association between serial measurements of ACA RI and continuously monitored Csat for 72 h after birth. This discrepancy can also be explained by the fact that NIRS may poorly reflect the exact cerebral blood flow, as it demonstrates cerebrovenous oxygenation. NIRS is not a direct indicator of perfusion adequacy because it provides information predominantly about the oxygen supply–demand balance [22]. Lower Csat was found to be significantly associated with severe IVH, which was noted in 15% of the observations performed by Altit et al. [14]. However, they did not find any differences in ACA Doppler parameters in scans between those with and without severe IVH. The authors emphasized that abnormally low Csat is associated with severe IVH. However, blood flow parameters were collected after IVH had already occurred, which may explain why ACA RI was not predictive of severe IVH. Therefore, whether ACA flow disturbances can predict severe IVH needs to be investigated. We measured ACA RI on the first postnatal day prior to IVH occurrence and evaluated it with the evolution of severe IVH. However, severe IVH occurred in only six infants in our cohort, and we did not find any association between ACA RI measurements and severe IVH (data not shown). In addition, no association was detected between the composite outcome and Doppler measurements at any time point. However, we found a significant association between ACA RI on the first postnatal day and early mortality. As shown in a review article by Lehtonen et al., early mortality is strongly associated with perinatal factors such as asphyxia and early-onset sepsis, especially in developing and underdeveloped countries [23]. Based on the findings above, we concluded that higher ACA RI values on the first postnatal day might be due to perinatal factors.

Some studies have suggested an association not only between early but also long-term neonatal outcomes and cerebral oxygenation [12, 13, 24]. Alderliesten et al. [12] found that low levels of cerebral oxygenation are associated with unfavorable cognitive outcomes. Chock et al. [13] reported a significant correlation between lower Csat and mortality or neuroradiographic injury. In our study, Csat in infants with adverse outcomes was low, similar to that reported by Chock et al., but we could not show a statistical difference when compared with infants without adverse outcomes. This may be attributed to the duration of NIRS monitoring, relatively lower incidence, and different definitions of adverse outcomes in our study.

A potential limitation of our study is that transfontanellar sonographic examination is operator-dependent and the amount of pressure applied among different infants might not be consistent. To maximize the accuracy and reproducibility of the technique, all measurements were taken by a neonatologist who is highly experienced in neuroimaging, and intraclass correlation showed a high consistency.

The strengths of our study are its prospective design, close neuromonitoring with serial cranial US studies, and early cerebral oxygenation monitoring in the critical window of life when infants are expected to have the greatest risk of brain injury. It is also one of the largest trials on very premature infants to date, providing further insight into cerebral monitoring with noninvasive tools.

## 5. Conclusions

Simultaneous monitoring of RI obtained from ACA and cerebral NIRS combined with routine cUS imaging may help detect clinically occult cerebrovascular complications earlier, monitor changes in hemodynamic status, and predict short- and long-term outcomes. We believe that our study is enlightening because the knowledge derived from the present study could serve as valuable data for future research to better understand the impact of perinatal conditions on cerebral hemodynamics and guide the management of very preterm infants with cerebral perfusion-oriented treatments.

## Figures and Tables

**Table:** Demographic and perinatal characteristics.

Gestational age, wk	28.3 ± 1.9
Birth weight, g	1090 ± 305
Head circumference, cm	26.7 ± 2.8
Male	45 (47)
Cesarean delivery	82 (85)
5-min Apgar	7 (6–8)
Small for gestational age	9 (9.5)
Maternal chorioamnionitis	6 (6)
Multiple birth	23 (24)
Preeclampsia	13 (14)
Antenatal steroids	66 (69)

Numbers are presented as mean ± SD, median (IQR) or n (%).
